# ‘It’s a can of worms’: understanding primary care practitioners’ behaviours in relation to HPV using the theoretical domains framework

**DOI:** 10.1186/1748-5908-7-73

**Published:** 2012-08-03

**Authors:** Lisa A McSherry, Stephan U Dombrowski, Jill J Francis, Judith Murphy, Cara M Martin, John J O’Leary, Linda Sharp

**Affiliations:** 1 National Cancer Registry, Building 6800 Cork Airport Business Park, Kinsale Road, Cork, Ireland; 2 Institute of Health and Society, Medical Faculty, Newcastle University, Baddiley-Clarke Building, Richardson Road, Newcastle, NE2 4AX, UK; 3 Aberdeen Health Psychology Group, University of Aberdeen, Health Sciences Building Foresterhill, Aberdeen, AB25 2ZD, Scotland; 4 Coombe Women and Infants University Hospital, Dolphin’s Barn, Dublin 8, Ireland

**Keywords:** Cervical screening, HPV, Clinical behaviours, GPs, Practice nurses, Primary care, TDF, Knowledge, Emotion, Social influences, Beliefs about capabilities, Beliefs about consequences

## Abstract

**Background:**

The relationship between infection with high-risk human papillomavirus (HPV) and cervical cancer is transforming cervical cancer prevention. HPV tests and vaccinations have recently become available. In Ireland, as elsewhere, primary care practitioners play a key role in prevention. ATHENS (A Trial of HPV Education and Support) aims to develop a theory-based intervention to support primary care practitioners in their HPV-related practice. This study, the first step in the intervention development process, aimed to: identify HPV-related clinical behaviours that the intervention will target; clarify general practitioners’ (GPs’) and practice nurses’ roles and responsibilities; and determine factors that potentially influence clinical behaviour. A secondary objective was to informally assess the utility of the Theoretical Domains Framework (TDF) in understanding clinical behaviours in an area with an evolving evidence-base.

**Methods:**

In-depth semi-structured telephone interviews were conducted with GPs and practice nurses. The topic guide, which contained open questions and HPV-related clinical scenarios, was developed through literature review and clinical experience. Interview transcripts were content-analysed using the TDF as the coding framework.

**Results:**

19 GPs and 14 practice nurses were interviewed. The major HPV-related clinical behaviours were: initiating a discussion about HPV infection with female patients; offering/recommending HPV vaccination to appropriate patients; and answering patients’ questions about HPV testing. While the responsibility for taking smears was considered a female role, both male and female practitioners dealt with HPV-related issues. All 12 theoretical domains arose in relation to HPV infection; the domains judged to be most important were: *knowledge*, *emotion*, *social influences, beliefs about capabilities* and *beliefs about consequences*. Eleven domains emerged in relation to HPV vaccination, with *beliefs about consequences*, *social influences*, *knowledge* and *environmental context and resources* judged to be the most important. Nine domains were relevant to HPV testing, with *knowledge* and *beliefs about capabilities* judged to be the most important.

**Conclusions:**

The findings confirm the need for an intervention to support primary care practitioners around HPV and suggest it should target a range of theoretical domains. The TDF proved valuable in analysing qualitative data collected using a topic guide not specifically designed to capture TDF domains and understanding clinical behaviours in an area with an evolving evidence-base.

## Background

More than 40 strains of human papillomavirus (HPV) are sexually transmitted and infect mucosal surfaces of the lower genital area [1]. Around 15 of these strains, including HPV16 and HPV18, are known as ‘high-risk’ types because they are a necessary cause of cervical cancer [2]. These high-risk types are common [3], asymptomatic, and cleared by most women in a few months; in some women, however, persistent infection(s) can lead to cervical pre-cancer and cancer [1].

It is generally accepted that, in the near future, cervical screening will be based on testing for infection with high-risk HPV types, rather than on conventional cytological smears [4-6]. Compared to smears, HPV testing (which can be conducted on residual smear samples) has higher negative predictive value and higher sensitivity for the detection of pre-cancer [7] making HPV-based screening likely to be effective [8,9] and cost-effective [10,11]. In addition, two prophylactic HPV vaccines have been developed [12]. For both vaccines, the vaccination schedule involves three doses at intervals within a six-month period and is most effective when administered before HPV exposure (*i.e.*, among the sexually naive). Organised vaccination, together with screening, could prevent most cervical cancers [13].

Ireland, which has a mixed public-private healthcare system, is at the forefront of this transformation in cervical cancer prevention. A national screening programme, CervicalCheck, was rolled-out in autumn 2008. The programme invites women aged 25 to 60 for a free smear in primary care every 3 to 5 years [14]. Prior to this, a woman could obtain a smear from her general practitioner (GP) or ‘Well Woman’ clinic for around €50. HPV testing is available through some practices and Well Woman clinics. CervicalCheck is currently introducing HPV testing in the follow-up of women treated for pre-cancer and is considering other uses, for example as a primary screening tool. HPV vaccination was licensed in males and females aged 9 to 26 in 2006, and can be purchased in primary care for around €600. In autumn 2010, a national schools-based vaccination programme started, providing free vaccination to girls aged around 12 [15]. These developments have been accompanied by changes in primary care, notably a move from single-doctor to multi-doctor practices supported by practice nurses. Traditionally, GPs were the primary smear takers, but practice nurses now play an important role in smear taking [16] and perform around one-half of the smears within CervicalCheck.

In addition to providing smear tests, GPs and practice nurses are likely to be key sources of information and advice for patients on HPV infection, vaccination, and testing. For women, their GPs’ attitude influences their own prevention behaviours [17,18]. Moreover, healthcare professionals’ compliance with, and encouragement of, HPV vaccination is crucial in achieving high vaccination rates [19]. Therefore, GPs’ and practice nurses’ clinical practices in relation to HPV will impact on the success of cervical cancer prevention strategies.

Little is known about what influences HPV-related clinical practice. Most research has focussed on practitioners’ knowledge, and while this is an important predictor of clinical behaviour, it is unlikely to be the sole influence [20,21]. A 2004 US family doctors’ survey found that substantial proportions were unaware of information on HPV infection relevant for patient counselling [22]. A 2007 survey of GPs in Ireland, using the same instrument (see Additional file 1), found lower knowledge levels than in the US survey [23], and important gaps in knowledge about HPV vaccination, consistent with findings from elsewhere [24-27]. More than 95% of GPs desired national guidelines or policy on HPV vaccination and testing. HPV infection knowledge predicted HPV vaccination intentions: GPs with higher knowledge scores were significantly more likely to be willing to vaccinate sexually naive girls aged under 16 than those with lower knowledge scores. A 2007 study among US family doctors, found the Theory of Planned Behaviour [28] variables intentions, subjective norms (perceptions about whether others approve of vaccination), and perceived behavioural control (perceptions about whether the decision to vaccinate is within the control of the doctor) influenced HPV vaccination behaviour [29]. No studies have investigated determinants of practice nurses’ clinical behaviours in this field.

ATHENS (A Trial of HPV Education and Support), which is being conducted under the umbrella of the CERVIVA research consortium (http://www.cerviva.ie) aims to develop a theory-based intervention to support primary care practitioners in their practice in relation to HPV infection, vaccination, and testing. The current study is the first step in this intervention development process. The primary aims were to: identify HPV-related clinical behaviours that the intervention will target; clarify roles and responsibilities of GPs and practice nurses in these areas; and determine what influences these clinical behaviours. Because little is known about practice, or potentially relevant psychological theories, we used qualitative methods to generate data with richness and depth, and analysed this using the Theoretical Domains Framework (TDF) [30]. As the TDF was originally developed to aid understanding of clinical behaviours around evidence-based guidelines, a secondary objective was to reflect the utility of the TDF in a way that may inform other researchers who are considering using it.

This article is one of a series documenting the development and use of the TDF to advance the science of implementation research. An overview of the articles contained in the series is provided in the introductory article [31].

## Methods

### Participants and recruitment

GPs and practice nurses working in Ireland were eligible to participate. GPs were recruited from a group of 145 participants in the 2007 survey [23], who were originally sampled from a national database and had indicated they were potentially willing to assist with further research. The group was diverse in terms of personal and practice characteristics and HPV infection knowledge and vaccination attitudes (in 2007). A purposive sample was recruited from this group, with strata defined in terms of variables that had been found in the 2007 survey to be strongly associated with attitudes towards smear taking and HPV vaccination, namely: gender, years since graduation, area of practice location, and HPV infection knowledge score in 2007 (questions shown in Additional file 1). GPs were approached by post, in batches in a random order, and invited to take part in an interview about their views and experiences of cervical cancer prevention. Those interested returned a reply slip, and the study co-ordinator (LAMcS) contacted them to arrange an interview. Non-respondents received up to two reminder letters. As interviews progressed, particular attention was paid to recruiting GPs in unfilled strata.

Since there is no national database of practice nurses, two approaches were used to identify potential participants. Firstly, all attendees at the annual national Irish Practice Nurses Association conference received a flyer and were invited to return this if they were interested in taking part. Secondly, invitation packs, compiled by the study team, were provided to professional development co-ordinators (PDCs) in six of the seven administrative areas across Ireland. The PDCs were each asked to select, at random, five nurses in their area to whom to send the packs. Nurses who returned the reply slip were contacted for interview. The only sampling stratum was area of practice and, as recruitment progressed, efforts were focussed on ensuring that nurses were recruited from urban and rural areas across the country.

Ethical approval was obtained from the ethics committee of the Irish College of General Practitioners. Participants provided informed consent.

### Procedures

Interviews were conducted by telephone by the study co-ordinator (LAMcS) and guided by a topic guide (Additional file 2). The topic guide was developed with input from a multidisciplinary team including investigators (LAMcS, LS, and SUD), the head of smear taker training at CervicalCheck, a practicing GP and practice nurse. It was informed by: literature review; a group discussion with the CervicalCheck smear taker training unit, who provide training sessions and a telephone helpline for GPs and practice nurses; discussions with the HPV vaccination team at the HSE National Immunisation Office and observation at one of their open meetings for GPs and practice nurses; and informal discussions with primary care practitioners. The guide included open questions and clinical scenarios designed to elicit information about HPV-related clinical behaviours, roles, and responsibilities in primary care, and drivers of clinical behaviours. It was organised in four clinical areas: cervical screening/smear taking, HPV infection, HPV vaccination, and HPV testing. The topic of cervical screening/smear taking was not the main focus of the study, but was included to set the context of the discussions about HPV. Participants were invited to discuss their experiences, views, barriers, and facilitators to practice, and support needs in each clinical area. The clinical scenarios covered HPV-related issues that could arise in practice and, potentially, present difficulties or challenges. Interviewees were asked to describe what they would do in each situation and how easy/difficult they would find it to handle. During the interview the interviewer chose which scenarios to present; these covered topics that had not already been raised by the interviewee, with the aim of eliciting as much information as possible.

Recruitment continued until new issues ceased to emerge for GPs and practice nurses separately. Interviews lasted 16 to 50 minutes and were audio-recorded; three participants declined to be recorded, and so detailed notes were taken instead. Recordings were transcribed verbatim and anonymised.

### Analysis

Content analysis was conducted following the framework analysis approach [32,33]. GP and practice nurse interviews were analysed together. The four clinical areas were considered separately. Two investigators (LAMcS, LS) read and re-read all transcripts, independently coded these, combined codes into subthemes and allocated these, and direct quotes from participants, to one of the 12 theoretical domains/themes of the TDF [30]. They held regular discussions to resolve disagreements and reach consensus and discussed uncertainties with a third investigator (JJF). To ensure analytical rigour, a second iteration of this process was performed, with re-review of transcripts to identify any important quotes or subthemes missed or misallocated. It was noted whether subthemes arose solely among GPs, practice nurses or both. The final synthesis and interpretation involved considering each theme/domain and subtheme in the context of the whole set of interviews. The strongest/dominant themes/domains were those: mentioned by most practitioners; where the most subthemes were identified; which were discussed at greatest length; and/or which were judged by the investigators to be invested with considerable intensity, passion, or sentiment by practitioners.

Although interviews covered cervical screening, the results reported here concentrate primarily on HPV infection, vaccination, and testing. Illustrative quotes have been provided to supplement narrative descriptions.

## Results

A total of 145 GPs were contacted, and telephone interviews were conducted with 19. Of the 30 practice nurses invited to take part through PDCs, ten were interviewed; a further four practice nurses, recruited at the annual conference, were also interviewed. Table 1 summarises participants’ characteristics.

**Table 1:** Characteristics of practitioners interviewed

		**GPs**	**Practice nurses**
**Gender**	Female	13	14
	Male	6	0
**Healthboard area**	HSE Mid-Eastern	4	3
	HSE North-Eastern	3	4
	HSE Southern	7	0
	HSE Western	5	7
**Location of practice**	City	6	6
	Other	13	8
**Solo GP practice**	Yes	6	7
	No	13	7
**Practice nurse**	Yes	16	-
	No	3	-
Years since graduation *	<10 years	2	n/a
	10-19 years	2	n/a
	20-39 years	14	n/a
HPV information knowledge score *^1^	High (11+)	5	n/a
	Medium (7-10)	10	n/a
	Low (<7)	3	n/a
HPV vaccine attitude *	Positive	10	n/a
	Negative / neutral	7	n/a

* Data from 2007 GP survey (Murphy *et al.*, 2008) [23].

^1^ Number of 13 factual questions about HPV infection that were answered correctly (Additional file 1).

n/a = not available.

### Clinical behaviours

A limited number of HPV-related clinical behaviours were identified. As regards HPV infection, the key behaviour was initiating a discussion on this topic with female patients. This was more often done if the practitioner had a relevant ‘opening,’ such as a patient with HPV reported on her smear result, or a patient presenting with genital warts. A few participants, mainly practice nurses, reported routinely mentioning HPV infection to women attending for smears. For HPV vaccination, three behaviours were identified: offering/recommending vaccination to appropriate patients; discussing vaccination when raised by a patient; and administering the vaccine. The first of these was the most common behaviour. HPV testing was very rare among practitioners and answering patients’ questions was the most important behaviour in this regard.

### Roles and responsibilities

Taking smears was considered a predominantly female role with responsibility falling on female GPs and practice nurses, who frequently stated that patients should always have the option of a female smear taker. Male GPs were less likely to perform smears and made comments like ‘I do an occasional one when a patient requests it.’

In contrast, HPV infection was discussed with patients by both male and female GPs and practice nurses. Similarly, responsibility for behaviours relating to HPV vaccination and HPV testing fell within the remit of both GPs and practice nurses. Sometimes, a GP described discussing HPV vaccination with patients and administering the first dose, then referring the patient to the practice nurse for the two subsequent doses.

### Factors that may influence clinical behaviours: HPV infection

All 12 theoretical domains played a role in relation to discussing HPV infection with patients (Table 2). *Memory attention and decision processes* was least frequently raised, being mentioned by a single participant. The strongest domains were *knowledge*, *emotion*, *social influences, beliefs about consequences* and *beliefs about capabilities.*

**Table 2:** Factors influencing behaviours related to HPV infection

**Theme / construct domain**	**Subtheme / specific belief**	Practitioner *		**Sample quote**
		**GPs**	**Practice nurses**	
**1. Knowledge**	Don't know enough about it	√	√	*‘Probably well the HPV now I have to say I’m not au fait as I say…’ - Practice nurse 5003*
	It’s an evolving area so it’s hard to keep up to speed	√	√	
	Not enough information available on it	-	√	
	Credibility of information sources	√	√	

**2. Skill**	Difficulty initiating a discussion on HPV infection with a patient	√	√	‘*We try and normalise the whole thing by saying, you know, if we took blood tests off everybody in the clinic you’d find something like 80% of us had evidence of HPV infection at some point in the past’ - GP 0140*
	Ability to ‘normalise’ HPV infection when talking to patients	√	√	
	Difficulty explaining HPV infection in a way that patients can understand	-	√	
**3. Social / professional role and identity**	Don’t want to pass judgement on patients’ sexual behaviours	-	√	*‘It’s a very mmm difficult situation and you don’t want to lay any blame - Practice nurse 5023*

**4. Beliefs about capabilities**	Difficulty dealing with awkward or sensitive situations	√	-	*‘There is difficulty because it’s often mmm it very much depends on the context. You know, if mmm if the relationship has broken down or if they have suspicions, they can be a very distressing for women to mmm ask those questions’ - GP 0058*
	Easier to discuss with patients who are open or interested	√	√	
	More likely to discuss HPV infection with patients if:			
	Already doing a smear	-	√	
	Patient asks for a prescription for ‘the pill’	√	-	
	Patient presents with genital warts	√	-	
**5. Beliefs about consequences**	When discussing HPV infection, patients might:			*‘You don’t know whether by highlighting it [HPV infection] that people might stop coming for smears in case it’s positive’ -Practice nurse 5002*
	Get embarrassed	√	√	
	Be put off having smears	-	√	
	Think they have a sexually transmitted disease	-	√	
	Think their partner is being unfaithful	-	√	

**6. Motivation and goals**	Don’t think it’s necessary to discuss HPV infection with patients	√	√	*‘I wouldn’t see any reason to [talk about HPV infection]’ -GP 0072*
	Discussing HPV infection with patients is important	√	-	

**7. Memory, attention and decision processes**	Topic is not at the forefront of the practitioner’s mind	√	-	*‘It’s not sort of, of hopping on to the top of my mind..’ -GP 0090*

**8. Environmental context and resources**	Don't have enough time for discussion	√	√	*‘I suppose maybe to have more aids in very simple descriptions in different languages…it’s simple things that I draw out and explain to people. I don’t have that. I suppose it’s something we need to get together maybe and work on’ - Practice nurse 5001*
	Need aids for discussion	-	√	
	Need leaflets about HPV to give to patients	-	√	
				
**9. Social influences**	Patients don't know anything about it	√	√	*‘I would say that most people coming in for cervical screening do not understand or even know the association of HPV to it… They do not associate HPV and cervical cancer in any way whatsoever’ - Practice nurse 5043*
	Patients don't want to know about it	√	√	
	Need for more publicity	√	-	
**10. Emotion**	Sensitive topic because it's about patients' sexual behaviour	√	√	*‘Well I mean the bottom line is that I think I’m em, along with most of my colleagues, are avoiding the subject. Because we know damn well that it’s about you know hhh you know behaviour you know sexual behaviour. And I mean it’s associated with sleeping with the wrong guy... I think we’re avoiding. I think you’re right, we’re avoiding the topic cos we don’t want to go near it because it’s a can of worms’ - GP 0034*
	The practitioner might get embarrassed	√	√	

**11. Behavioural regulation**	Recognising opportunities to raise topic with patients	√	√	*‘I try to work it [HPV infection] in sometimes when I’m talking to them initially about contraception and if they’re using the pill and stuff like that.’ - GP 0026*
	Having aids for the discussion available	-	√	
	Having leaflets available to give to patients	√	√	
	Having a clear plan of what to say	√	√	

**12. Nature of the behaviour**	Don't routinely bring HPV infection up with patients	√	√	*‘It’s not something that I on a regular basis discuss with people…’ - Practice nurse 5043*

* √ = Mentioned by at least one practitioner.

- = Not mentioned by any practitioners.

The *knowledge* domain had several layers. First, practitioners described a general lack of knowledge and need for more information. Second, the evidence-base was perceived as rapidly evolving, and practitioners reported difficulty keeping up-to-date. Third, practitioners questioned the credibility of some information sources. For example, they noted that much of the information on HPV came from pharmaceutical companies, and they considered it to be biased. In contrast, participants expressed confidence in CervicalCheck as an information source not just as regards smears, but also in relation to HPV. As regards *emotion*, there was a general belief that the whole area of HPV is ‘sensitive’ and ‘awkward’ as it relates to sexual behaviour. Practitioners were concerned about patient embarrassment and, for some, their own embarrassment. The strength of the emotions involved led practitioners to adopt coping strategies such as being ‘careful’ in what they said, ‘tiptoeing’ around the topic, or avoiding it altogether. Patient lack of interest or knowledge was given as another reason for not discussing HPV infection (*social influences*). In addition, some practitioners were reluctant to raise the topic because they felt it might discourage women from having smears (*beliefs about consequences*). In relation to *beliefs about capabilities*, both GPs and practice nurses indicated that they found it difficult to initiate a discussion on HPV infection without some kind of ‘opening,’ such as a direct question from the patient.

As regards the *skill* domain, GPs observed that they found it difficult to explain HPV in a way that patients could understand. Practice nurses observed that discussing HPV infection could be seen as passing judgement on an individual’s sexual behaviour, which would be professionally inappropriate (*social/professional role and identity).* In terms of *motivation and goals*, some GPs questioned the need to discuss HPV infection with patients at all because the infection can resolve on its own and/or there is no treatment. A lack of time in consultations and lack of aids for discussion were noted in regard to *environmental context and resources*. Some practitioners recognised opportunities to raise the topic with patients (*e.g.*, when a patient presented with genital warts or asked for a prescription for the contraceptive pill) (*behavioural regulation*). Overall, however HPV infection was not widely discussed with patients (*nature of the behaviour*).

### Factors that may influence clinical behaviours: HPV vaccination

The only theoretical domain that did not emerge in relation to HPV vaccination was *memory, attention and decision processes* (Table 3). The dominant domains were *beliefs about consequences*, *social influences*, *knowledge* and *environmental context and resources*.

**Table 3:** Factors influencing behaviours related to HPV vaccination

**Theme / construct domain**	**Subtheme / specific belief**	Practitioner *		**Sample quote**
		**GPs**	**Practice nurses**	
**1. Knowledge**	Not enough information available about it	-	√	*‘I suppose the big difficulty with it really is mmm what to do with girls who are mmm maybe that little bit older... You’re not quite sure if they have had sexual encounters or not and whether it’s still worthwhile giving it or not’ - GP 0058*
	Rapidly changing area	-	√	
	Uncertainty over how long the protection afforded by vaccination will last	√	√	
	Uncertainty over whether to vaccinate older / sexually active girls or women	√	-	
	Uncertainty over whether to vaccinate boys	√	√	
	Most of the information comes from pharmaceutical companies and is viewed as biased	√	√	

**2. Skill**	Difficulty of dealing with a consultation where both mother and daughter are present (and/or mother asking about vaccination for daughter)	√	√	*‘It’s quite a tricky consultation [when mother and daughter are both present] and you’re very aware of everybody’s, the confidentiality issues for the girl, mmm for you know that mum who’s come with her, for all of the type of thing’ - Practice nurse 5001*
	Difficulty assessing whether a patient has been sexually active	√	√	

**3. Social / professional role and identity**	Feel out of touch (because HPV vaccination programme is being delivered through schools)	-	√	*‘You see it’s very difficult and I find it’s quite difficult since most of my patients are GMS*^*§*^*and in many of these houses there’ll be a few girls, and I know the people can’t afford the bloomin stuff ..’ - GP 0090*
				
**4. Beliefs about capabilities**	Practitioners are not comfortable discussing HPV vaccination	√	-	*‘It’s a bit too new probably compared to some of the other, you know the baby vaccines that are out like they’re going donkeys years now and they seem fairly safe. I’m sure the other two [HPV vaccines] are as well, but I can’t guarantee it’ - Practice nurse 5020*
	The vaccine is too new to be considered in primary care	-	√	
				
**5. Beliefs about consequences**	HPV vaccination can cause serious side effects	√	√	*‘Are you allowing them to be more sexually active by giving them the vaccine. .. I must admit personally speaking initially when I first saw I was oh my goodness you know girls will become more promiscuous’ - Practice nurse 5040*
	Vaccination might encourage promiscuity	√	√	
	Belief that vaccine is effective	√	√	
	Belief that vaccine is safe	√	√	
**6. Motivation and goals**	Don't think HPV vaccination is necessary	√	-	*‘I don’t know now...how much it’s [HPV vaccination] warranted’ - GP 0086*
**7. Memory, attention and decision processes**	-	-	-	-

**8. Environmental context and resources**	The cost is very high	√	√	*‘I think the price is astronomical. And outrageous…’ - GP 0034*
	It would involve a lot of extra work for GPs to provide HPV vaccination	√	-	
	HPV vaccine is not stocked in the surgery	√	-	

**9. Social influences**	If practitioner has a daughter they would vaccinate her	√	√	*‘If it was daughter I’d do it [vaccinate her]’ - GP 0016*
	Patients don't know anything about HPV vaccination	√	√	
	Parents don't want to know that their children are sexually active	√	√	
	There has been a lot of negative publicity about HPV vaccination	√	√	

**10. Emotion**	Nervousness about managing an HPV vaccination consultation when both mother and daughter are present	√	√	*‘That can be a little bit of a minefield there especially if you’ve got the mother and daughter in front of you’ - GP 0026*
**11. Behavioural regulation**	Sourcing vaccine at a cheaper price	√	√	*‘We got together as a group… and we sourced it as cheaply as we could because we bought it as a group so we got a group discount’ - Practice nurse 5001*
	Having written information available to provide to patients	√	√	
	Having posters advertising that the vaccine is available in the surgery / waiting rooms	√	√	
**12. Nature of the behaviour**	Don't see patients in the relevant age group	√	√	*‘I actually don’t see very many young you know, at the ages where I could see them around 12 or 13… I so rarely see children now’ - GP 0092*

* √ = Mentioned by at least one practitioner.

- = Not mentioned by any practitioners.

§ Patients who have access to free GP appointments and prescriptions by having a means-tested medical card.

Some practitioners believed that HPV vaccination was effective and safe, but others had concerns about side effects (*beliefs about consequences*). For example, some raised the case of a schoolgirl in the UK who died shortly after receiving the vaccine in 2009. In general, practitioners who had daughters were more likely to have a positive attitude towards HPV vaccination (*social influences*). Others perceived that parents would think that HPV vaccination would encourage promiscuity in their children, and negative media publicity was cited by some as a reason for not discussing it with patients. The same *knowledge* issues arose for HPV vaccination as for HPV infection (*i.e.*, lack of knowledge, need for more information, and credibility of information sources). The evolving evidence base emerged as especially challenging in relation to practice. For example, practitioners expressed considerable uncertainty over whether to vaccinate sexually active girls. Few were clear or certain of what to do when faced with this, and other related situations, in the clinic. Others were certain about what they would do and why, but their reported practice conflicted with current evidence. In relation to *environmental context and resources*, the major barrier to practitioners recommending HPV vaccination to patients was cost; for most practitioners this was their primary concern about vaccination.

Linked to cost, practitioners perceived that there was an ethical difficulty associated with recommending to patients something that the practitioner knew patients could not afford (*social/professional role and identity*). Some practitioners mentioned that they were attempting to provide the HPV vaccine at a cheaper price (*e.g.*, by buying in bulk, or forming a buying consortium with other practices) (*behavioural regulation*). Some practitioners had experienced a consultation about HPV vaccination where both mother and daughter were present and this was described as particularly ‘tricky’ to manage *(skill)* and as a ‘minefield’ (*emotion*). In addition, some noted that it could be difficult to determine whether a patient had been sexually active (*skill*). In terms of *beliefs about capabilities,* concerns were expressed about the ‘newness’ of the vaccines. Finally, some practitioners did not see patients in the relevant age group (*nature of the behaviour*) while others did not feel that HPV vaccination was necessary (*motivation and goals*).

### Factors that may influence clinical behaviours: HPV testing

Nine of the 12 theoretical domains emerged in relation to HPV testing. The dominant ones were *knowledge* and *beliefs about capabilities* (Table 4). Fewer subthemes surfaced for HPV testing than for the other clinical areas.

**Table 4:** Factors influencing behaviours related to HPV testing

**Theme / construct domain**	**Subtheme / specific belief**	Practitioner *		**Sample quote**
		**GPs**	**Practice nurses**	
**1. Knowledge**	Not enough information available about it	√	√	*‘I didn’t even know that it [HPV testing] was available… Whatever it is whether it’s a urine or a swab… Just don’t have the knowledge at all’ - Practice nurse 5003*
	Don't know anything about it	√	√	
**2. Skill**	-	-	-	-
**3. Social / professional role and identity**	Topic not covered in practitioner's training	-	√	*‘That wasn’t brought up inmy training or any of our updates’ - Practice nurse 5018*
				
**4. Beliefs about capabilities**	The test is too new to implement in routine practice	√	√	*‘We don’t have any algorithm for the management of people with HPV’ - GP 0140*

	Would be more likely to discuss HPV testing if:			
	There were guidelines / management algorithm	√	√	
	There was a reliable source to whom practitioners can refer questions	√	-	

**5. Beliefs about consequences**	HPV testing provides no clinical benefit	√	√	*‘I’m not sure there’s any point, I can’t see the point in it really. It doesn’t seem to add an awful lot to [the treatment plan for women with cervical cancer], like given that you’re going to come up with a positive or negative test result to just HPV in geneeral mmm it doesn’t seem to add anything’ - GP 0133*
				
**6. Motivation and goals**	HPV testing would be useful in primary care	√	-	*‘I think it would be good to provide HPV testing’ - GP 0129*
				
**7. Memory, attention and decision processes**	-	-	-	-

**8. Environmental context and resources**	HPV testing costs too much	√	√	*‘From what I understand it’s very expensive’ - Practice nurse 5018*

				
**9. Social influences**	More publicity is needed to encourage women to have a HPV test	√	-	*‘For the public to come forward [for HPV testing] a media campaign is always very good because if they read something in the daily papers they’ll take heed of it. They won’t take heed of us advising them a lot’ - GP 0086*


**10. Emotion**	The practitioner could be embarrassed by not having answers to patients questions about HPV testing	√	-	*‘It’d be very difficult because it’d be mortifying not to have the answers straight away’ - GP 0034*

**11. Behavioural regulation**	HPV testing is uncommon in Ireland	-	-	-
**12. Nature of the behaviour**	HPV testing is uncommon in Ireland	-	√	*‘I don’t think it’s done an awful lot in Ireland’ - Practice nurse 5020*
				

* √ = Mentioned by at least one practitioner.

- = Not mentioned by any practitioners.

In terms of *knowledge*, there was very limited awareness of HPV testing and both GPs and practice nurses were unclear about what testing involved, how it was done and whether it was available in Ireland. Practitioners observed that there was a need for HPV testing guidelines or management algorithms, and these would make them more likely to consider offering testing *(beliefs about capabilities*).

Some practitioners who were aware of HPV testing felt that there was uncertainty around its clinical benefit (*beliefs about consequences). Emotion* emerged in relation to professional embarrassment associated with not being able to answer patients’ questions about HPV testing.

## Discussion

### Roles and responsibilities

A striking finding of the study was that male GPs had moved away from responsibility for smear taking; this was seen to be a predominantly female role. Furthermore, in recruiting to the study, it was particularly difficult to get male GPs to participate because they saw cervical cancer prevention as outside their remit. This means that practice nurses and female GPs may have more opportunity to raise HPV infection, vaccination, and testing with patients (*e.g.*, while they are conducting smears). However, patients may ask a male GP questions about HPV, outside of the screening setting. Thus, it is important that male GPs keep up-to-date with developments around HPV and, specifically in relation to ATHENS, both male and female GPs should be targeted by any intervention in the area of HPV-related clinical practice.

### Factors that may influence clinical behaviours

All theoretical domains emerged as influences on clinical practice. Only one—memory attention and decision processes—did not play a significant role but was mentioned by one participant in relation to one clinical behaviour. Even for HPV testing, which was very uncommon, nine of the domains emerged as potential influences on practice. This perhaps reflects how complex practitioners consider the topic of HPV to be. Some domains surfaced more strongly and these are discussed below.

### Emotion

Various studies show that women consider HPV a sensitive topic because it is associated with sexual behaviour [34,35]. This was echoed in our study: practitioners observed that HPV infection is a sensitive topic for patients and gave this as a reason for not raising it in consultations. While it is likely that this theme would also emerge elsewhere, it is possible that it may be particularly strong in Ireland because of cultural and social norms around discussing or admitting sexual behaviour. For example, 2008 research among women in Ireland found that there was a considerable stigma attached to having smears because it was seen to be an admission of sexual activity [17,18]. Interestingly, the issue of sensitivity around HPV did not seem to be limited to patients; practitioners’ comments about HPV infection also revealed emotional influences. For example, they frequently talked about the difficulty of raising the subject with patients and the underlying tone of some interviews was tentative and awkward. One might expect that healthcare professionals would have ample experience of dealing with sensitive topics. Therefore, it is unclear whether there is something particularly challenging about HPV itself or whether practitioners’ emotions are influenced by their lack of knowledge, concerns about their ability to deal with patients’ reactions (*skills*), or other issues. Further research would be valuable to investigate the relationship between relevant theoretical constructs.

### Social influences

Practitioners often stated that they did not discuss HPV with patients because they believed that patients either did not know anything, or did not want to know anything, about it. Studies in various counties have shown that there is limited knowledge about HPV amongst women [36-40]. However, a lack of knowledge is not universal, and a population survey of women in Ireland conducted in 2010 (O’Connor *et al.* 2010; personal communication) found that 44% had heard of HPV infection and 55% had heard of HPV vaccination. In addition, qualitative research among women in Ireland in 2008 found that, on learning about the link between HPV and cervical cancer, women were shocked, angry, and felt that this was a ‘secret’ that the medical community had kept from them [18,19]. Women also wanted to know more about HPV. Thus, it may be counterproductive for practitioners to assume that women have not heard of HPV or are not motivated to discuss it. Our findings also suggest that influencing practitioners’ perceptions about patient influences (*e.g.*, a lack of desire to know about HPV) might increase the behaviour. We also found that practitioners’ reported that HPV infection is difficult to explain in a way that patients understand (*skill*), and there is insufficient time in consultations (*environmental context and resources*) suggesting that other factors interact with perceived *social influences* in relation to discussing HPV infection. It is also possible that reasons cited by practitioners for not discussing HPV infection may mask a reluctance of practitioners themselves to discuss the topic.

### Beliefs about consequences

*Beliefs about consequences* was important in relation to both HPV infection and vaccination. For HPV infection, only negative consequences were raised, while for HPV vaccination, both positive and negative consequences emerged, with practitioners tending to discuss one aspect or the other. Of note, practitioners who raised concerns regarding vaccination safety were generally unaware that the death of a schoolgirl in the UK following vaccination was subsequently found to be unrelated to the vaccination [41]. The polarized views about the consequences of vaccination may reflect the fact that the HPV evidence-base is still evolving, particularly in relation to long-term efficacy and safety [12]. However, the fact that this domain was important suggests that influencing practitioners’ *beliefs about consequences* might influence their behaviours in relation to HPV.

### Knowledge

Previous research found gaps in doctors’ HPV infection and vaccination knowledge [22,23]. This study confirms these and shows that primary care nurses also have limitations in their knowledge. Knowledge gaps were reported directly by practitioners, in their own words, and also became apparent through their responses to the clinical scenarios, meaning that practitioners varied in the extent to which they perceived that their knowledge was limited. Practitioners also described difficulties in keeping abreast of the clinical evidence; this has been identified as a barrier to primary care practice and patient care in other clinical areas [42-44].

Knowledge limitations, and the uncertainty expressed about current evidence, are perhaps not surprising given that the HPV vaccination programme in Ireland started only recently, data continues to emerge from the HPV vaccination trials [12], HPV testing is both relatively new and rare in primary care, and no clinical guidelines are in place. However, high-risk HPV infection was established as a necessary cause of cervical cancer a decade ago [2], and the key features of HPV infection have been clear for several years. For example, the same answers would be correct nowadays for all of the questions contained in the instrument developed by Jain *et al.* in 2004 to assess practitioners’ HPV infection knowledge [22]. Hence, there are probably fewer uncertainties in the evidence-base around HPV infection, and it is not as rapidly evolving, as practitioners perceive.

It was noteworthy that CervicalCheck was seen as a trusted source for information on issues beyond smear taking (*i.e.*, around HPV) given that CervicalCheck had not produced formal advice or practice guidelines in relation to HPV in primary care. While this finding suggests that practitioners hold CervicalCheck in high esteem, it probably also reflects the fact that HPV information is perceived not to be available elsewhere, or at least not from a source that practitioners view as trustworthy.

For many practitioners, uncertainty around evidence flowed into an uncertainty about what to do in practice. Our analysis further suggested that this might have influenced how practitioners felt about the clinical areas and this, in turn, impacted on their practice (*e.g.*, leading to avoidance). We also found a contrast between *knowledge* and *beliefs about capabilities*. Some GPs especially were high in self-efficacy although it was clear from the interviews that they lacked knowledge. This suggests knowledge to be a necessary intervention target, but shows that it is unlikely to influence clinical practice if other relevant key variables are not taken into account. Further research thus needs to establish how knowledge and other potentially relevant factors suggested in this study act together to influence behaviour.

### Reflections on using the TDF

This study was the first stage in the development of an intervention, a process being guided by the MRC Framework for the Development of Complex Interventions [45,46]. Some investigators have observed that developing an intervention is time-consuming [47] and all studies, including ATHENS, have resource limitations. We applied the TDF, within a framework analysis approach [32], to analyse qualitative data collected using a topic guide not specifically based on the TDF domains. One of the main advantages of this was that the analysis was focused and efficient. The structured approach afforded by using the TDF to pre-define themes facilitated cross-checking the allocation of subthemes to themes and reaching analytical consensus. No influences on practitioners’ behaviours arose in the interviews that could not be classified according to the TDF, underlining the comprehensiveness of the framework. The possibility cannot be excluded that if a less structured analytical approach had been taken other aspects of clinical practice and experience might have emerged, but this risk is minimised by the wide range of psychological constructs included in the TDF. Equally, it is possible that a different analytical approach would have resulted in the themes being labelled or interpreted somewhat differently; this, however, could apply to almost all qualitative studies. A further strength of using the TDF was that our analysis was not prematurely confined to a particular psychological theory. This was, of course, a major rationale for the development of the TDF [30]. In our context, we had little *a priori* information on which to base the selection of appropriate theories. In view of the number of theoretical construct domains that emerged in relation to each clinical area, it was clear that using the TDF had strengthened the study: had we focussed from the outset on one or two theories (*e.g.*, Theory of Planned Behaviour) [29], it is likely that we would have missed important influences on clinical behaviour and this would have impacted on the likely effectiveness of any intervention subsequently developed.

The evidence base for HPV testing in cervical screening and efficacy of HPV vaccination continues to develop and to date there are no guidelines on HPV in primary care in Ireland. One potential limitation of the TDF is that it was designed for use in situations in which clear evidence-based clinical practice guidelines are in place. It has previously been suggested that the theoretical domains might be of less utility in situations without clear-cut guidelines or in which the evidence base is somewhat uncertain, because the effect of potential predictors might be overwhelmed by variations in attitudes [48]. This did not seem to be an issue in our context. Although there were variations in practice and attitudes, between nine and 12 of the domains were relevant to each clinical behaviour.

A further potential limitation is that the TDF does not specify relationships between the construct domains. Our synthesis and interpretation suggested that there may be links between psychological constructs in influencing behaviour (*e.g.*, between *beliefs about capabilities* and *knowledge*), but the TDF does not enable formal investigation of these links. Nor were we able to determine, other than in a qualitative way, which of the domains were likely to be the most important drivers of clinical behaviour. However, these limitations were in part a function of the qualitative study design, rather than the TDF *per se*. This study was the first step in an intervention development process and was intended to be hypothesis generating rather than hypothesis testing. Hence, we chose qualitative methods to provide a detailed picture of roles and responsibilities in clinical practice around cervical cancer prevention and to identify which domains may play a role. The next stage in the development process is a quantitative study in which we will determine the frequency of the behaviours of interest, identify the most important predictors of behaviours and investigate inter-relationships between domains and constructs predicting these behaviours. We have used the results reported here to design a questionnaire for this quantitative study. This proved to be a very efficient method of questionnaire development. Questions were included covering the domains and subthemes that were reported by more than one practitioner; this helped in focusing and limiting the length of the questionnaire, and should reduce the possibility of redundant questions. The language used by interview participants was used to form question stems, which should enhance face validity. The questionnaire study is currently under way.

## Conclusions

The findings of this study confirm the need for an intervention to support primary care practitioners in their HPV-related practice. This intervention should target both male and female practitioners and should be directed towards discussing HPV infection with female patients, offering or recommending HPV vaccination to appropriate patients and answering patients’ questions around HPV testing. Such an intervention is more likely to be effective if it is aimed at a range of theoretical domains. The TDF proved valuable in analysing qualitative data collected using a topic guide not specifically designed to capture TDF domains, and understanding clinical behaviours in an area with an evolving evidence-base.

## Abbreviations

ATHENS: A Trial of HPV education and support; GPs: General practitioners; HPV: Human papillomavirus; PDC: Practice development co-ordinator; TDF: Theoretical domains framework.

## Competing interests

LAMcS, SUD, JJF, JM and LS have no competing interests. CMM and JJO’L have received funding from Hologic for evaluating the CERVISTA HPV test.

## Authors' contributions

LAMcS was involved in design of this study, recruited participants and conducted interviews, undertook analysis and interpretation and wrote the initial and subsequent drafts of the manuscript. SUD was involved in design of this study and contributed to the development of the topic guide and drafting of the manuscript. JJF was involved in the analysis and interpretation of the findings and contributed to the drafting of the manuscript. JM was involved in the design and development of ATHENS and commented on the manuscript. CMM was involved in the design and development of ATHENS and commented on the manuscript. JJO’L was involved in the design and development of ATHENS and commented on the manuscript. LS was involved in the design and development of ATHENS and the design of this study, undertook data analysis and interpretation and assisted LAMcS in drafting the manuscript. All authors read and approved the final manuscript.

## Authors’ information

The ATHENS group comprises:

Investigators: LAMcS, SUD, JJF, JM, CMM, JJO’L and LS

Steering Group: Dr Brenda Corcoran, Ms Ann McGill, Ms Eileen O’Donovan, Dr Eamonn Shanahan, Dr Alan Smith, and investigators as listed above

Intervention Development Group: Ms Carol McNamara, Ms Louise McKee, Dr Genevieve McGuire, LAMcS, SUD and LS.

## Supplementary Material

Additional file 1 **GPs’ knowledge of HPV infection. **Questions used in 2007 GP survey in Ireland to compute HPV knowledge score. Click here for file

Additional file 2 Interview topic guide. Click here for file
