# Supplementary material for: ‘It’s a can of worms’: understanding primary care practitioners’ behaviours in relation to HPV using the theoretical domains framework
Source: Implement Sci. 2012 Aug 3;7:73. doi: 10.1186/1748-5908-7-73 (PMC3523072; doi:10.1186/1748-5908-7-73)
Supplement: Additional file 1 — GPs’ knowledge of HPV infection. Questions used in 2007 GP survey in Ireland to compute HPV knowledge score. [file 1748-5908-7-73-S1.doc]

Additional file 1: GPs’ knowledge of HPV infection. Questions used in 2007 GP survey in Ireland to compute HPV knowledge score.

GPs were invited to indicate whether they believed each statement to be “true” or “false”, and were awarded a score of 1 for each correct response and 0 for each incorrect response. The maximum possible score was therefore 13.

| 1. Genital HPV infection is fairly common in sexually active adults^1^ |
| --- |
| 1. A person with genital HPV infection may never show symptoms or signs of infection^1^ |
| 1. Most genital HPV infections may be cleared without medical intervention^1^ |
| 1. Persistent genital HPV infection in women increases risk of cervical dysplasia and cervical cancer^1^ |
| 1. Genital HPV infection in men increases risk of penile and other anogenital cancers^1^ |
| 1. Treatment of cervical dysplasia/cancer does not always permanently eliminate the causative infection^1^* |
| 1. Genital HPV infection causes external anogenital warts^1^ |
| 1. Genital HPV types usually associated with external anogenital warts differ from types usually associated with cervical dysplasia and cervical cancer^1^ |
| 1. External anogenital warts do not increase the risk of cancer at the same site where the warts are located^1^* |
| 1. Treatment of external anogenital warts does not always permanently eliminate the causative infection^1^* |
| 1. Available tests and procedures cannot determine the duration of a patient’s genital HPV infection^1^* |
| 1. Risk of contracting genital HPV infection decreases with increasing age* |
| 1. Condom use does not provide effective protection against genital HPV infection** |

* = These statements were phrased as false statements in the questionnaire but have been reworded here as true statements.

** = Currently there is inconclusive medical evidence to support the statement. At the time of the 2007 survey, this statement was considered to be true.

**^1^** Original source of questions: Jain N, Irwin K, Montano D, Kasprzyk D, Carlin L, Freeman C, Barnes R, Christian J, Wolters C: **Family Physician's Knowledge of Genital Human Papillomavirus (HPV) Infection and HPV-related Conditions.** *Fam Med* *2006*, **38**(7): 483-9.
