# Supplementary material for: ‘It’s a can of worms’: understanding primary care practitioners’ behaviours in relation to HPV using the theoretical domains framework
Source: Implement Sci. 2012 Aug 3;7:73. doi: 10.1186/1748-5908-7-73 (PMC3523072; doi:10.1186/1748-5908-7-73)
Supplement: Additional file 2 — Interview topic guide. [file 1748-5908-7-73-S2.docx]

Additional file 2: interview topic guide

**Q.** Can you tell me a few details about the practice that you work in?

***Clinical area: Cervical screening/smear taking***

**Q.** Can you tell me a bit about the set-up for smear testing in your practice?

**CS.**

1. *A 44 year old woman comes in for a consultation about her diabetes. At the end of the consultation, just as she is leaving the room, she says “I saw an ad for CervicalCheck yesterday”. And she asks you “Do I need a smear?”.*

or

1. *A 28 year old woman, a patient in your practice, tells you that she has recently attended an STI Clinic and is being treated for genital warts, which she has been told are HPV related. She says that the specialist told her to go to her GP practice for a smear test.*

or

1. *A 52 year old woman, who is a new patient in your practice, presents with a CervicalCheck invitation for a smear test. She tells you she had a hysterectomy about 15 years ago, and asks whether she needs to have a smear test.*

What would you do in this situation?

On a scale from 1 (not difficult at all) to 7 (extremely difficult), how difficult would it be to handle this situation?

***Clinical area: HPV infection***

**Q.** Do patients ask you about HPV infection?

**Q.** Do you initiate discussions on HPV infection with patients?

**Q**. What do you do once you have started a discussion about HPV infection?

**Q.** What can make it difficult for you to talk to patients about HPV infections?

**Q**. What can make it easier for you to talk to patients about HPV infections?

**CS.**

1. *A 51 year old woman in your practice has an ASC-US smear result. She asks if it is true that this result is due to her having ‘got’ the HPV virus.*

or

1. *A 51 year old woman has a low grade LSIL smear result, after a history of several normal smears. She asks you “Why have I suddenly got this now?”*

or

1. *A 29 year old woman has a low grade LSIL result. She has read that this is caused by HPV and she wants to know how she can get rid of the infection.*

What would you do in this situation?

On a scale from 1 (not difficult at all) to 7 (extremely difficult), how difficult would it be to handle this situation?

***Clinical area: HPV vaccination***

**Q.** Do you provide HPV vaccination in your practice?

**Q.** Does your practice proactively encourage HPV vaccination?

**Q.** Have you ever had patients ask you about HPV vaccination?

**Q.** What can make it difficult for you to talk to patients about HPV infections?

**Q.** What can make it easier for you to talk to patients about HPV infections?

**CS.** *A 36 year old mother, who is a patient in your practice, asks you if her 17 year old girl should get the HPV vaccine.*

What would you do in this situation?

On a scale from 1 (not difficult at all) to 7 (extremely difficult), how difficult would it be to handle this situation?

## *Clinical area: HPV testing*

**Q.** What are your thoughts on providing HPV testing?

**Q.** Do you provide HPV testing in your practice? / Would you consider providing it?

**Q.** Have any of your patients ever requested a HPV test?

**Q.** What can make it difficult for you to talk to patients about HPV infections?

**Q.** What can make it easier for you to talk to patients about HPV infections?

**CS.** *A 33 year old woman presents a HPV test result from a UK general practice, which is positive for high-risk HPV. She wants to know how this test result will impact on her cervical screening interval.*

What would you do in this situation?

On a scale from 1 (not difficult at all) to 7 (extremely difficult), how difficult would it be to handle this situation?

## *Support needs*

**Q.** How do you keep up-to-date with cervical screening and HPV?

**Q.** Do you think GPs/practice nurses in Ireland need more support around cervical screening and HPV?

## *Other issues*

**Q.** Is there anything else related to smear testing or HPV in primary care that you feel is important?

Q=major question stem; appropriate follow-up questions depended on the interviewees response

CS=clinical scenario; where multiple scenarios are presented, one was selected during the interview to cover issues not already raised by the interviewee.
